# A Nano LC-MALDI Mass Spectrometry Droplet Interface for the Analysis of Complex Protein Samples

**DOI:** 10.1371/journal.pone.0063087

**Published:** 2013-05-09

**Authors:** Fiona Pereira, Xize Niu, Andrew J. deMello

**Affiliations:** 1 Institute for Chemical and Bioengineering, Department of Chemistry and Applied Biosciences, ETH Zürich, Zurich, Switzerland; 2 Engineering and the Environment, and Institute for Life Sciences, University of Southampton, Highfield, Southampton, England; Institute of Enzymology of the Hungarian Academy of Science, Hungary

## Abstract

The integration of matrix-assisted laser desorption ionization (MALDI) mass spectrometry with an upstream analytical separations (such as liquid chromatography and electrophoresis) has opened up new opportunities for the automated investigation of complex protein and peptide mixtures. The ability to efficiently analyze complex proteomic mixtures in this manner is primarily determined by the ability to preserve spatial discrimination of sample components as they leave the separation column. Current interfacing methods are problematic in this respect since minimum fraction volumes are limited to several microliters. Herein we show for the first time an LC-MALDI interface based on the formation, processing and destruction of a segmented flow. The interface consists of a droplet-generator to fractionate LC effluent into nL-volume droplets and a deposition probe that transfers the sample (and MALDI matrix) onto a conventional MALDI-MS target. The efficacy of the method is demonstrated through the analysis of Trypsin digests of both BSA and Cytochrome C, with a 50% enhancement in analytical performance when compared to conventional interface technology.

## Introduction

Mass spectrometry (MS) is a powerful analytical technique capable of assaying a wide range of chemical and biological systems in a label free manner. Of particular note is matrix-assisted laser desorption/ionization (MALDI) mass spectrometry. This MS variant, introduced in the late 1980s by Hillenkamp and Karas, provides for the efficient and soft ionization of large and often fragile biomolecules [1], [2]. For these reasons MALDI-MS has become a popular and efficient tool in the analysis of proteins and peptides [3] ^.^ Unfortunately MS methods are unable to extract useful (and quantitative) information when applied to complex protein mixtures. To address the challenges of sample complexity, mass spectrometers are often coupled to one or more separation techniques either in an *on-line* or *off-line* format and often MS is the last step in the proteomic analytical process [4]. Liquid chromatography (LC) and capillary electrophoresis (CE), in their various embodiments, are high efficiency separation techniques, and are among the primary methods that have been hyphenated to mass spectrometers [5], [6]. Not surprisingly, the interface between LC and MALDI-MS plays an important role and has been studied intensively [7], [8]. In the *off-line* format, LC effluent (consisting of multiple, spatially separated components) is channelled to a tee-junction where it is mixed with an appropriate MALDI matrix and then spotted onto the target using contact or non-contact deposition [9], [10].*On-line* MALDI has also been achieved using spray, continuous flow or mechanical interfaces [11], of which continuous flow and mechanical interfaces have been applied in microfluidic formats [7], [12].

Conventional methods of fraction collection after an analytical separation (and before introduction into the mass spectrometer) typically involve the transfer of eluting bands into sample vials or microwell plates. This is hugely problematic in terms of maintaining high theoretical plate numbers and component resolution since minimum fraction volumes are limited to several microliters [13]. To this end, both Niu *et al.* [14] and Edgar *et al.* [15] have recently demonstrated the use of segmented liquid flows as an efficient tool for collecting and compartmentalizing effluent from both macro- and micro-scale separation systems. In the study by Niu and co-workers, droplet generation after a (first dimension) LC separation is followed by depletion of the continuous (oil) phase and droplet merging prior to a (second dimension) electrophoretic separation. Importantly, the interface is passive in its operation, employing a pillar array to actively extract the oil phase. This ensures negligible transfer of oil and complete transfer of droplet contents into the separation channel. The ability to partition peaks originating from a first separation dimension into a stream of droplets is significant since it ensures that chemical or biological information (resolution) is not lost during transfer.

To date, most droplet interfaced LC-MS research has focussed on the interfacing of segmented flows with electrospray ionization mass spectrometry (ESI-MS). For example, Fidalgo *et al.* first reported the integration of ESI-MS with droplet-based flows [16]. In this study droplets flowing parallel to an adjacent aqueous stream are transferred into the aqueous stream (and then onto the ESI emitter) via application of an electric field orthogonal to the flow. Although the approach was successful in allowing MS analysis of individual droplets, Taylor dispersion of the analyte after phase transfer leads to significant dilution and thus sub-optimal detection limits. Kelly *et al.* further demonstrated dilution-free droplet to MS interface via hydrophilic surface coating [17]. More recently, Zhu and Fang described the extraction of aqueous droplets using a hydrophilic ‘tongue’ [18]. Here sample is siphoned into a hydrophilic carrier channel from a hydrophobic, segmented flow channel prior to delivery into a monolithic ESI emitter. This approach was successful in monitoring peptide alkylation but also suffered from sample dilution post extraction. Finally, Li and co-workers recently described a commercial set up for LC effluent fraction collection and integration to offline ESI-MS [19]. Interestingly, the authors report the deleterious effect of the oil continuous phase on the ionisation process and resulting MS signals. Accordingly, they describe a passive method for oil removal where the carrier phase collects at the emitter tip, migrates away from it and is then siphoned away in Teflon tubing. These studies confirm that efficient oil removal is a critical parameter when delivering segmented flows to downstream MS processing. Despite the obvious gains in terms of analytical efficiency there have been few reports describing the interfacing of segmented flows with MALDI-MS. Hatakeyama *et al.* first reported the use of droplets as microreactors followed by MALDI-MS analysis of droplet contents via contact deposition onto standard MALDI target plates [20]. Interestingly, the authors did not remove the oil phase prior to droplet deposition and unfortunately did not assess the effects of oil on the ionization process. For a traditional LC-MALDI-MS system, where the effluent is mixed with MALDI matrix and is deposited on a target plate, the probability of band broadening and remixing is higher than LC-ESI, where ESI emitter can be integrated directly to the LC separation column [21].

**Figure 1:**
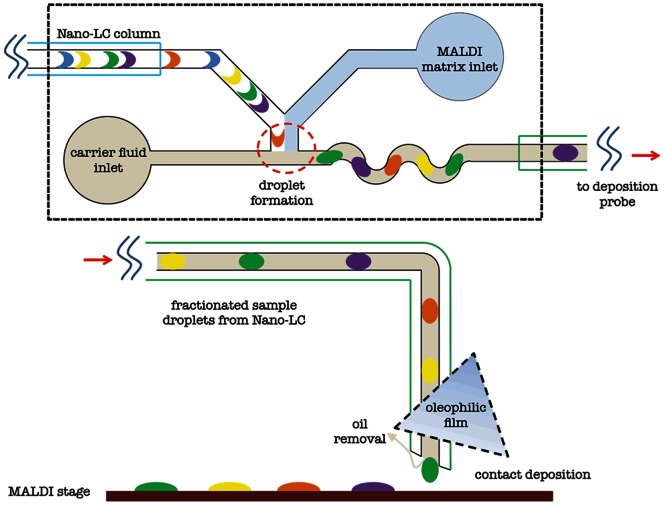
Schematic of the Nano-LC MALDI-MS droplet interface device. (a) Water-in-oil droplets are generated at a microfluidic T-junction having two aqueous inlets, one oil inlet and an outlet channel. The LC effluent is transferred into the microfluidic device via a fused silica capillary. The second aqueous inlet is used to introduce MALDI matrix in a controlled manner. LC effluent and MALDI matrix meet at the T-junction and are delivered into the oil stream where they are broken into droplets due to shear forces. (b) The deposition probe consists of a 200 µm *i.d.* Teflon tubing and an oleophilic membrane. Droplets generated in the interface device are transported to the MALDI target via the Teflon tubing. The PTFE membrane extracts the continuous oil phase leaving the aqueous droplet suspended at the tip of the tubing. This droplet is then directly spotted onto the MALDI target by contact deposition.

Herein, we present for the first time an LC-MALDI interface based on the formation and processing of segmented flows. Significantly, the interface is passive in action and allows efficient removal of the continuous phase prior to deposition onto unmodified MALDI targets, without recourse to spatial (wettability) patterning of the surface. In simple terms, the interface employs a microstructure composed of hydrophobic and oleophilic membrane to absorb and remove oil at the tip of the deposition probe. The device consists of two parts: a droplet generation microdevice that fractionates the LC effluent into droplets and a deposition probe that is used to transfer and spot the sample onto the MALDI-MS target (Figure 1). The device can be positioned distal to the outlet of the LC separation column ensuring that the resolution gained by the separation is preserved once the analytes are fractionated into nL-volume droplets. Importantly, the LC eluent can be combined with MALDI matrix prior to droplet formation, allowing droplet delivery to the target in a “MALDI-ready” format.

## Materials and Methods

### Interface Device and Deposition Probe

The nano liquid chromatography to MALDI mass spectrometry interface was designed in two parts; a schematic of each part is illustrated in Figure 1. The first was a two inlet T-junction droplet generation microdevice used to mix the LC effluent with MALDI matrix and then fractionate the mixture into droplets. The second part was a deposition device that delivers and deposits the droplets onto the MALDI target and extracts oil from the segmented flow. The interface device was fabricated in Polydimethylsiloxane (PDMS) using standard soft lithographic techniques. The channels accommodating the liquid chromatography capillary (30 µm internal diameter (I.D.) and 100 µm outer diameter (O.D.)) and Teflon tubing (200 µm I.D. and 320 µm O.D.) were 300 µm wide and 150 µm high. Narrower channel dimensions were used for droplet generation and mixing. The 300 µm wide LC inlet channel narrows to 100 µm (which is the size of all other channels in the device), while the height of the channels remains at 150 µm. The two other inlets in the microdevice were used to introduce MALDI matrix and FC-40 oil. FC-40 oil is a perfluorinated, colourless, odourless and thermostable lubricant that is widely used in droplet formation in microfluidics. Liquids were driven through the device at specified volumetric flow rates (0.5 µl/minute) using syringe pumps (PHD Programmable 2000, Harvard Apparatus, UK).

To construct the deposition device, we coated the tubing ends with Aquapel prior to insertion. Aquapel is a commercially available water repellent agent (PPG Industries, Pittsburgh, PA) to render a hydrophobic surface. The arrangement of tubing and film ensures that oil is absorbed as it exits the tubing whilst the aqueous droplets collect at the tip of the tubing. Deposition onto the MALDI plate was carried out by contacting the tip of the probe to the surface of the plate either for a specific amount of time or until the aqueous droplet falls under the effects of gravity. For all protein calibration experiments, an acrylic holder with the XYZ stage was used to move the probe to the designated position of the MALDI plate.

### Sample Preparation

The interface device and deposition probe were tested prior to attachment to the nano-LC using protein stock solutions. Bovine Serum Albumin (BSA, 7 mg/ml), Cytochrome C (5.5 mg/ml), and Hen Egg white Lysozyme (18 mg/ml) were each prepared in a 0.1% trifluoroacetic acid (TFA) solution (Sigma Aldrich, UK) and approximately 175 µg, 137 µg and 450 µg of protein injected in the Nano HPLC respectively. The MALDI matrix used for the protein samples was Sinapinic acid (Sigma Aldrich, UK). It was prepared at a concentration of 12.5 mg/ml in 45% acetonitrile (ACN), 45% ethanol and 10% of an aqueous solution of 0.1% TFA. Two commercially available peptide digests were used to test the microfluidic interface and compare it to the automated LC-MALDI sample spotter, the Probot™ (Dionex Corporation, Amsterdam). Lyophilised Trypsin digests of Cytochrome C (Dionex Corporation, Amsterdam) and Bovine Serum Albumin (New England BioLabs, USA) were reconstituted in 200 µl of 0.1% TFA prior to LC analysis and mixed with MALDI matrix alpha-Cyano-4-hydroxycinnamic acid (HCCA).

### Mass Spectrometry Instrumentation

Mass spectrometry was carried out on two different instruments. Mass analysis of proteins was carried out on a Micromass® MALDI micro MX™ mass spectrometer (Waters, Manchester, UK). Positively charged ions in the mass range of 5000–150000 Daltons (Da) were analysed in the linear mode. A hundred single-shot spectra were gathered manually in groups of 10 from random spots within each sample well on the MALDI plate. These spectra were summed and processed using the smoothing and base line correction functions provided in the Mass Lynx software. Mass analysis of positively charged peptide ions was carried out on the MALDI 4800 (Applied Biosystems, California, USA). Positively charged ions in the mass range of 500–5000 Daltons were analysed automatically in the reflector mode. A thousand single-shot spectra were gathered in groups of 25 from random spots within each sample well on the MALDI plate.

### Nano-LC Instrument and Automated MALDI Spotter

The LC instrument used to test the microfluidic interface was the UltiMate™ 3000 system (Dionex Corporation, Amsterdam) consisting of a degasser, an autosampler (WPS 3000), a thermostated flow manager module (FLM 3000), a UV flow cell (UVD 3000) and Micro-pumps (LPG 3000). The mobile phases used for the reverse phase separation were Buffer A: 0.1% trifluoroacetic acid and 2% acetonitrile v/v and Buffer B: 0.1% TFA and 90% acetonitrile v/v. The peptide digest samples were loaded on to a PepMap™ 300 µm×5 mm C_18_ reverse phase trapping column and then eluted into the PepMap™ 75 µm×150 mm C_18_ analytical separation column in back flush mode at a flow rate of 0.4 µl/minute. The separation was performed across a gradient of 0–60% Buffer B and completed in 36 minutes. The peptides eluted off the analytical column were initially passed through the UV flow cell to ascertain resolution between peaks and then run into the Probot™ spotter.

Detection was carried out at 220 nm. The length of capillary joining the analytical separation column to the UV flow cell was approximately 62 cm and the length of capillary connecting the flow cell to the spotter was 72 cm. Once required separation resolution was obtained, the following samples were run directly into the spotter without routeing them through the UV flow cell. The length of capillary connecting the separation column to the automated spotter was 97 cm, while in the case of droplet interfaced Nano LC-MS, the droplet generation microdevice is attached 12 cm from the analytical separation column, and therefore totally 85 cm of continuous fluidic conduit was replaced with the droplet transferral.

## Results and Discussion

Initial experiments involved calibration of droplet generation and deposition using native proteins. Cross contamination between deposited droplets along with the effect of the continuous oil on the MALDI-MS signal and matrix crystallisation were also investigated (Figures S1 and S2 and Tables S1 and S2). Droplets were generated using a standard T-junction microdevice. In all experiments the aqueous phase consists of a mixture of protein solution and MALDI matrix while the carrier phase was pure FC-40 oil. Protein and matrix solutions were combined in a controlled and laminar fashion 500 microns upstream of the T-junction where droplets were formed. Once generated, the droplets were manoeuvred through three channel winds, which aided mixing of the two aqueous components via chaotic advection [22], and were then directly transferred into tubing that leads to the deposition probe.

**Figure 2:**
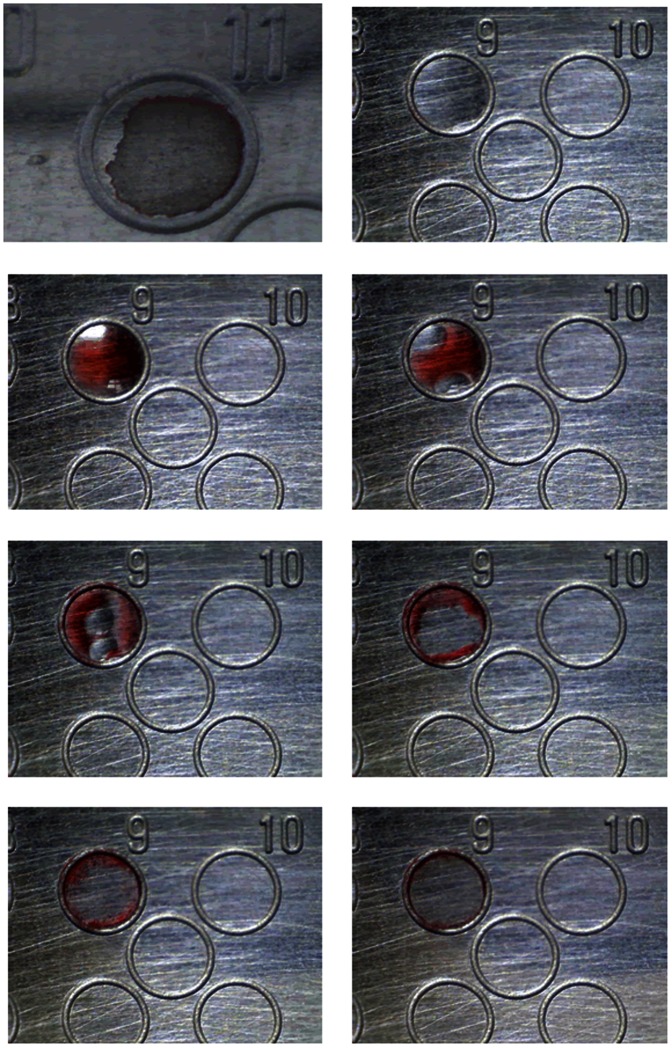
Effect of FC-40 oil on the crystallisation of HCCA. (a) Typical crystal surface formed using 1 µm of the matrix prepared in the dried drop format, (b) Addition of 0.5 µm of FC-40 oil onto the top left hand side well, (c) Addition of 0.5 µm dye loaded matrix onto the same well after 3 seconds, (d) after 5 seconds, (e) after 7 seconds, (f) after 10 seconds, (g) after 13 seconds and (h) after 20 seconds.

It is recognised that the quality of matrix crystallisation and the MALDI-MS spectra obtained for large biopolymers can be affected by the matrix solvent composition, the matrix-sample preparation procedure and the analyte-to-matrix ratio 23,24. Accordingly, when using droplets to fractionate and deliver MALDI-ready LC effluent, the aqueous phase should ideally be extracted from the carrier oil phase before it is spotted onto the MALDI target. In the current work, this is achieved using a deposition probe that combines an oleophilic film material with Polytetrafluoroethylene (PTFE) tubing as shown in Figure 1. Figure 2 (and Video S1) illustrates the effects of oil on HCCA matrix crystallisation, with the standard dried-droplet format being shown in Figure 2a. Initially, the sample-loaded matrix sinks beneath the FC-40 oil (Figure 2c). This is followed by the generation of bubbles in the oil due evaporation of the acetonitrile and ethanol solvents (Figure 2d–e). Bubble formation moves the matrix and analyte within the sample well and finally deposits it at the well edge (Figure 2f–h). Significantly, oil is driven from the sample well during evaporation of the solvent but returns in part, once the solvent has evaporated. This distributes sample and matrix around the well edge with a partial or entire covering of oil. The effect is volume dependent and exacerbated by increasing the oil-to-matrix-analyte solution ratio. In the presence of oil the sample dries in approximately 120 seconds compared to less than 60 seconds when using the standard dried-droplet format [23]. Similar behaviour was also noted for Sinapinic acid (data not shown), the other matrix used in this work.

**Figure 3:**
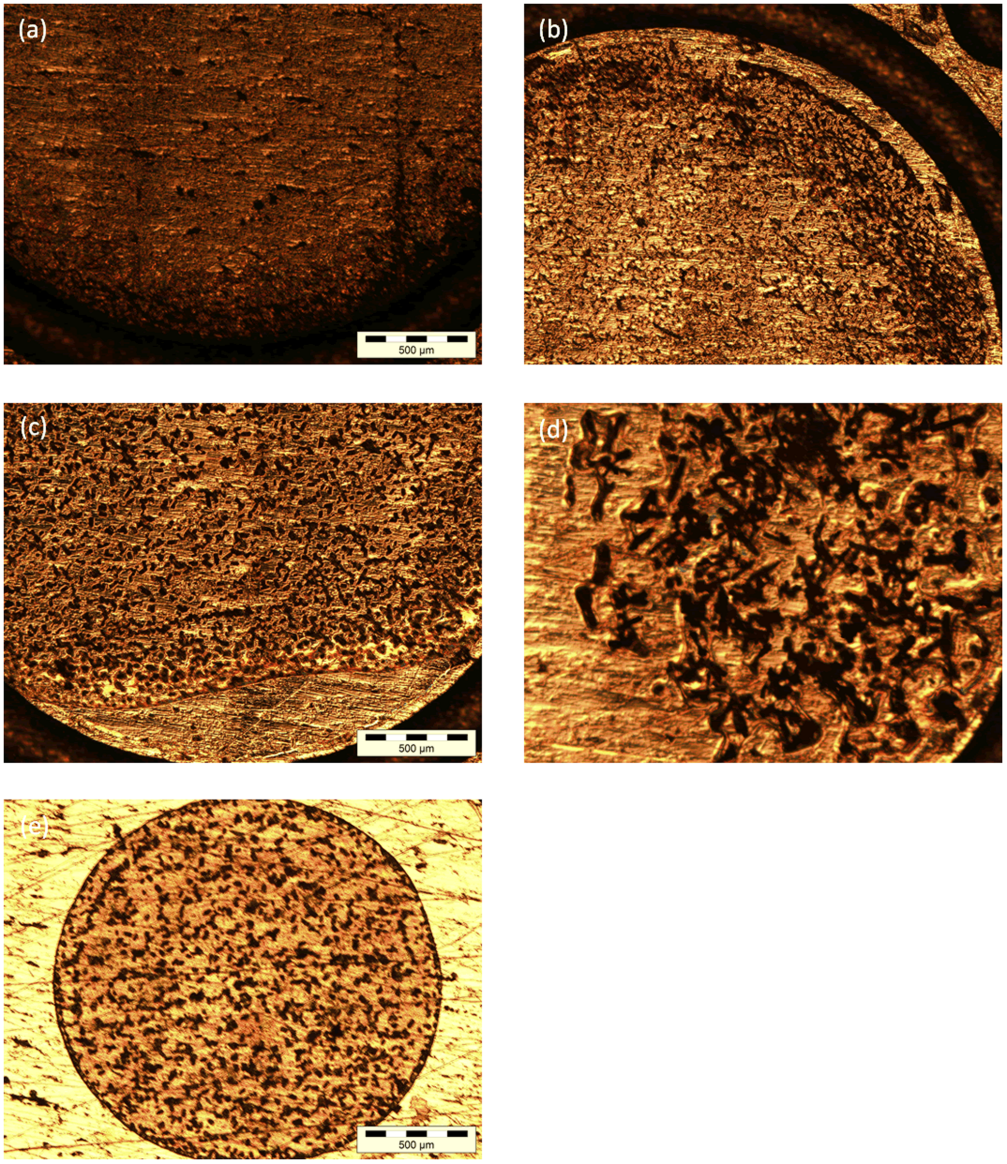
Sinapinic acid matrix crystallisation using the dried drop and droplet deposition method. (a) Matrix without oil, (b) Matrix in the presence of FC-40 oil. (c) BSA and matrix in the absence of FC-40 oil (d) BSA and matrix in the presence of FC-40 oil and (e) matrix crystals formed when a sample is spotted using the droplet deposition probe.

Compared to the traditional dried-drop method, crystallisation of matrix under oil has different features. These were imaged at x5 magnification to assess deposition quality. Figure 3 shows images of crystals from Sinapinic acid (Figure 3a and b), Sinapinic acid with BSA (Figure 3c,d) and Sinapinic acid and BSA spotted using the deposition device (Figure 3e). Results obtained for the HCCA matrix are detailed in Figure S3. Both matrices exhibit larger crystals when the matrix or the matrix-analyte solution is crystallised under oil. For both matrices, the presence of oil leads to the formation of an inhomogeneous surface with large matrix crystals distributed randomly over the surface of the sample. This makes signal acquisition complicated, and indeed edging may lead to little or no signal if measurements are acquired in an automated fashion (where the mass spectrometer is pre-set to examine and average results from the entire area within the sample well). Significantly, the sample spotted using the deposition probe (Figure 3e) consists of crystals of a similar size to those prepared using the traditional dried-drop method. Furthermore, mass spectra extracted from samples deposited in the presence of FC-40 oil were irreproducible. Depending on the matrix distribution on the MALDI plate, manually acquired signals reported protein peaks in regions where matrix crystals were observed. However, under automatic acquisition, extremely low (or no) signal intensity was observed for 50% of the spotted samples. Accordingly, no correlation between signal intensity and sample concentration was observed. Indeed, Cohen and Chait have previously compared the effect of dried-drop and rapid and slow crystallisation methods on the quality of MALDI mass spectra using two different HCCA matrix solvent systems [23]. In both cases they found that the adoption of slower crystallisation procedures leads to a mass discrimination effect, where only the higher molecular weight peptides are observed in the MALDI spectra, while the dried-drop and rapid crystallisation methods show the peptide peaks expected.

In the current experiments, 337.2 nm radiation is absorbed by the matrix. This causes sublimation and protein ionisation into the gas vapour phase above the matrix surface, without direct absorption by the analyte (thus preventing thermal degradation). Measurements indicated that FC-40 oil has a negligible molar extinction coefficient between 328 and 400 nm, therefore its presence over the sample does not affect the amount of laser energy reaching the matrix. However, vaporisation of the photo-excited matrix with attached analyte is likely to be impeded by the presence of the oil layer, due to trapping of gas phase ions. Additionally, cooling of the matrix prior to sublimation due to the transfer of thermal energy to the oil layer may also impede desorption and ionisation.

**Figure 4:**
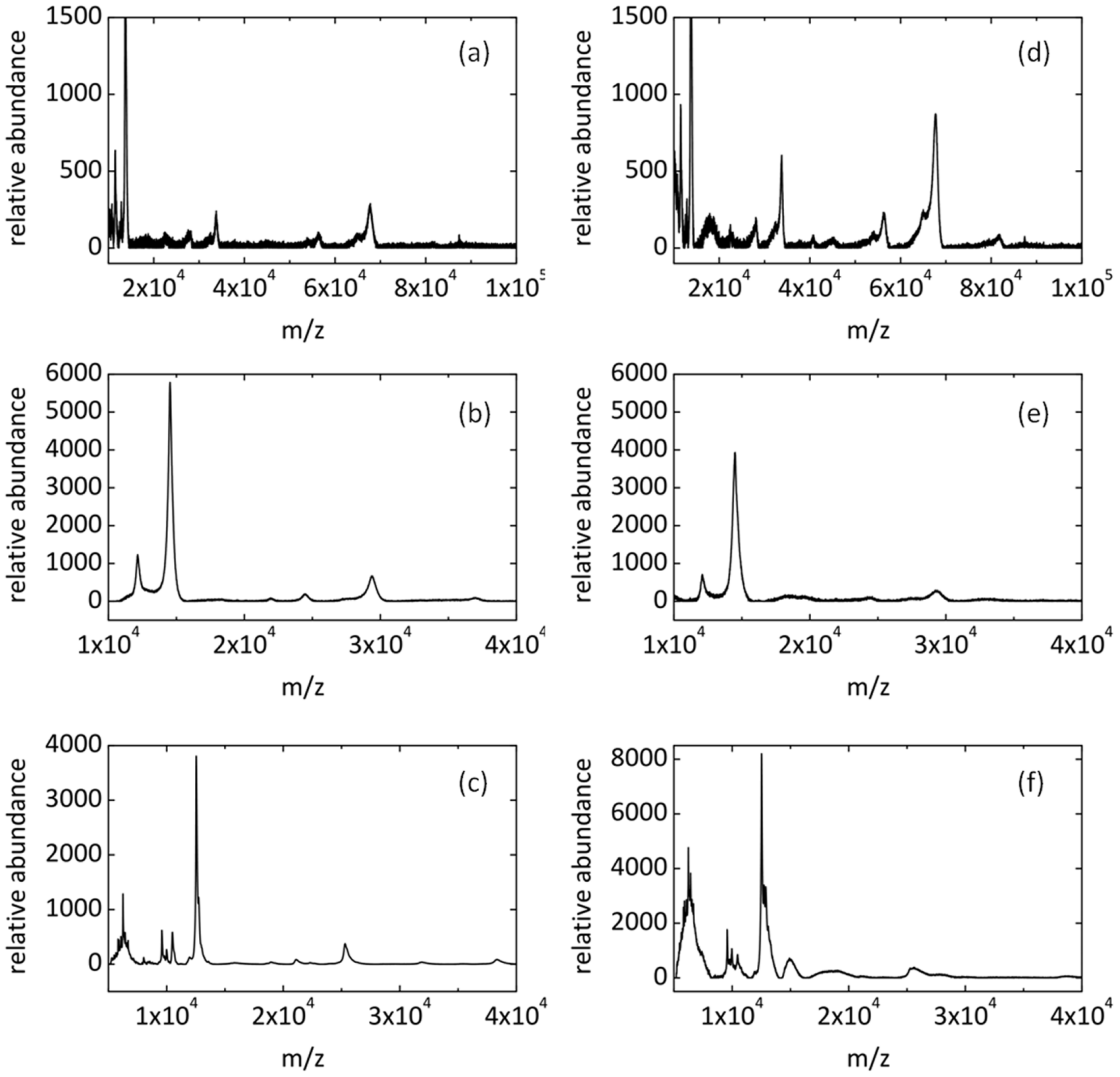
MALDI MS spectra of BSA, Cytochrome C and Lysozyme. (a–c) Spectra obtained from samples spotted using the traditional dried drop technique. (d–f) Spectra obtained from samples spotted using the microfluidic interface and deposition probe. Proteins were prepared at stock concentrations and diluted 1∶1 in a Sinapinic acid matrix prepared at a concentration of 12.5 mg/ml in 45% acetonitrile, 45% ethanol and 10% (0.1%) trifluoroacetic acid.

In initial experiments a fused silica capillary (ID 30 microns and OD 100 microns) was attached to a syringe loaded with a stock protein solution. Aqueous input flow rates were varied to control the size of the droplets entering the deposition probe. Figure 4 presents mass spectra of Bovine Serum Albumin, Lysozyme and Cytochrome C samples spotted using the traditional dried-drop technique (Figure 4a,c,e) and prepared using the droplet deposition probe (Figure 4b,d,f). All protein samples spotted using the deposition probe exhibit peaks in the expected mass-to-charge range and closely match control samples spotted using the traditional dried-drop technique. A close correspondence between signal intensities for each deposition method indicates that the developed interface and probe are highly effective in generating droplets and separating the continuous oil phase from the aqueous droplets during deposition.

To demonstrate the power of the interface, LC-separated peptides were mixed on-line with MALDI matrix (HCCA at 12.5 mg/ml) in a 1∶1 ratio and then segmented into 12 nL-volume droplets. Droplets were subsequently transported to the deposition probe and spotted onto an unmodified MALDI target. Nano-LC eluate was also spotted using a commercial spotter (Probot™ Dionex) as a control. In this case the HCCA matrix was prepared at 3.0 mg/ml in acetonitrile and water (1∶1 ratio) and deposited using contact deposition. A lower than customary concentration of matrix was used on the commercial machine as the matrix had been shown to precipitate out of solution at higher concentrations. To enable comparison between our deposition probe and the automated commercial spotter, a spotting frequency of 4 spots/minute was employed for all experiments. Both methods of spotting generated approximately 90 spots per sample. It should be noted that each deposited spot contains approximately 17 droplets. Accordingly in the current experiments resolution of droplet interface is compromised due to the recombination of multiple input droplets.

**Figure 5:**
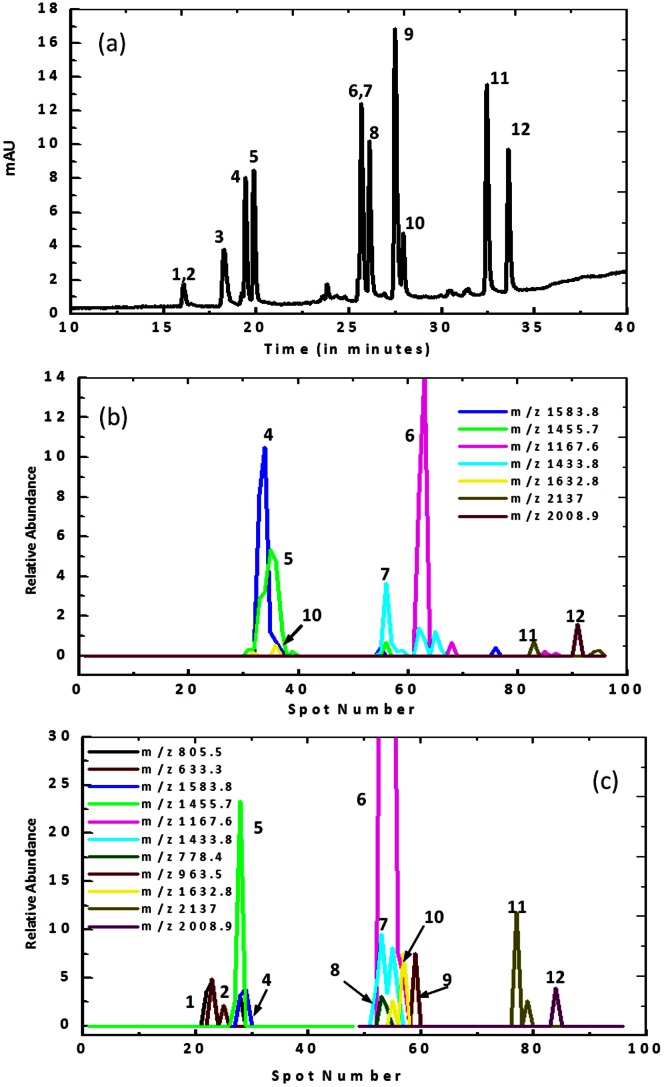
LC–MALDI MS analysis of peptides generated from Trypsin digested Cytochrome C. (a) The LC separation profile of the Cytochrome C digest. (b) and (c) detail the mass spectrometry results using a commercial spotter and using the droplet deposition probe respectively. The mass spectrometry results were plotted as relative peptide abundance versus the spot number. Table S3 compares the results from both spotters.

Two Trypsin digested proteins, Cytochrome C and BSA, were used to assess the performance of the deposition probe. Separated peptides were passed initially through the UV flow cell of the liquid chromatograph to generate a chromatogram, after which the eluted peptides were channelled directly to the appropriate spotter. The Trypsin digested Cytochrome C sample produced twelve peptides when separated by Nano-liquid chromatography (Figure 5a). The elution order is provided by the manufacturer for all twelve components as listed in Table S3. All peptides except peaks 1 and 2 and 6 and 7 were resolved. A single peptide band exiting the deposition probe can be deposited in a single spot or across several sample spots depending on its width. Figure 5b and c show the relative abundance of the separated peptides as a function of spot number across the MALDI target. It can be seen that commercial spotter is able to discriminate and identify 7 out of the 12 components (having mass values within 0.1 of the manufacturer’s guidelines), with the lower mass peptides not being observed. In comparison, the droplet-interfaced deposition probe allows discrimination of 11 out of 12 components with the same mass tolerance of 0.1. In this situation, the only peptide not observed is the smallest expected fragment having a mass of 633.5 Da (peak 3 in Figure 5a).

The chromatographic UV profile of Cytochrome C can be divided into 3 groups of eluted peaks, i.e. 1–5, 6–10 and 11 and 12. Importantly, integration of the droplet interface with the deposition probe provides a MALDI-MS result that preserves the resolution obtained during this chromatographic separation. The three distinct groups were maintained at the peak level, i.e. peaks 1–5, 6–10 and peaks 11 and 12 were spotted in the same sequence in which they were eluted from the chromatographic column (with the exception of peak 3). There was a small loss in resolution between bands in each group; however increasing the spotting frequency and reducing the distance between the chromatographic column and the droplet generation microdevice is expected to ameliorate this problem. Comparatively, the results originating from the automated spotter show more severe remixing between bands. The loss-in-resolution in the automated spotter can be attributed to the longer tubing length and continuous pipe flow of the Nano-LC effluent, inducing Taylor dispersion which will reduce the resolution between the resolved peptide bands. Indeed such kind of loss-in-resolution and remixing was studied in various continuous microfluidic devices [25], [26]. Moreover, in the automated spotter, sample adhesion to the capillary surface might increase cross contamination between eluting bands.

**Figure 6:**
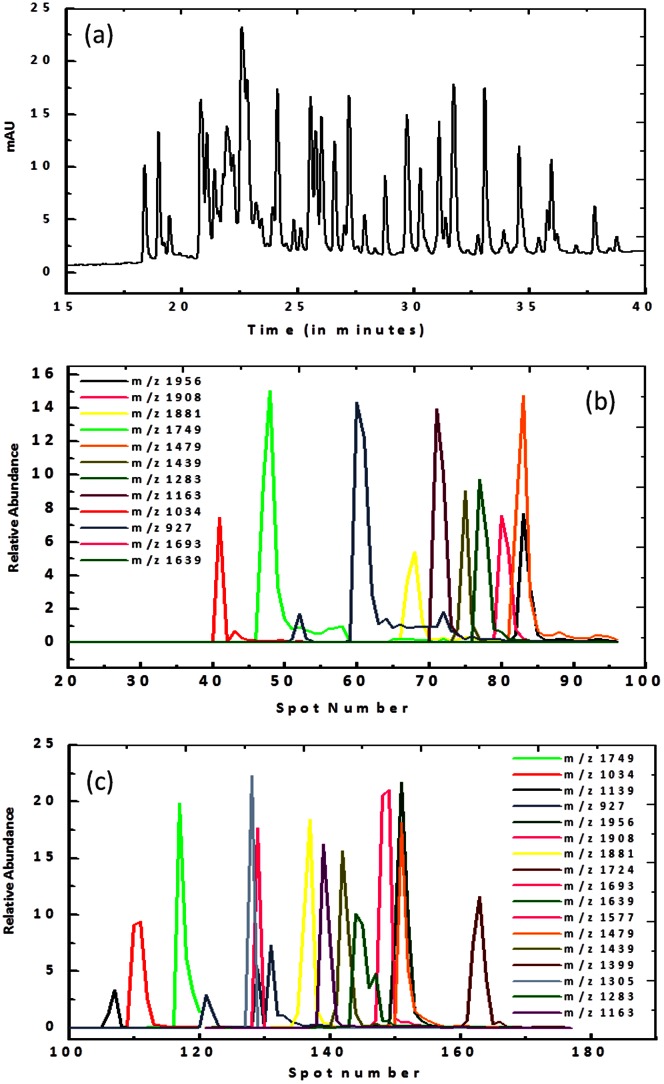
LC–MALDI MS analysis of peptides generated from Trypsin digested Bovine Serum Albumin. (a) The LC separation profile of the BSA digest. (b) and (c) detail the mass spectrometry results using a commercial spotter and using the deposition probe respectively. The mass spectrometry results were plotted as relative peptide abundance versus the spot number. Table S4 compares the results from each spotter and lists the mono-isotopic mass of each peak.

To further assess the performance of the deposition probe a more complex sample, Trypsin digested BSA was analysed. The Nano-LC separation (Figure 6a) shows numerous peaks that elute between 15 and 40 minutes; however, the supplier only provides mass information for 22 of these peaks. The MS data for the commercial spotter (Figure 6b) reports 12 peptides, while the deposition probe reports 19 distinct peptides. Table S4 lists the peaks found using each spotting format, both with a mass tolerance of 0.1. Three peptides at 544, 688 and 2492 Da were not observed using either spotting methods. This is most probably due to either the fact that the LC effluent was not sampled for the entire duration of the run and that the matrix obscured the signal from the smaller peptides. A further seven peaks of known mass were not observed when using the automated spotter which may be due to ion suppression by co-eluting peaks, because the tailing effect can be clearly seen in Figure 6b. Consequently, the spotted sample may contain several peptides, as well as closely eluting fragments leading to competitive ionisation and loss of signal.

### Conclusions

We have demonstrated a novel droplet-based interface between Nano–liquid chromatography and matrix assisted laser desorption ionisation mass spectrometry. Compartmentalisation of LC effluent into nL-volume droplets provides a facile and direct method of preserving chromatographic information between analytical methods. On-line combination of analytical samples with the MALDI matrix can be performed in a continuous manner and the resulting compartmentalised mixtures can be transported to conventional MALDI targets via the deposition probe. Furthermore, oil extraction can be achieved with single droplet recovery from the segmented flow. Compared to existing techniques, this process of oil removal is immensely enabling given that droplet contents are not diluted on transferral to the mass spectrometer. This unique feature is critical in a number of real-world applications, since oil adversely affects both the matrix-analyte crystallisation and desorption/ionisation process. MS analysis of native proteins shows that the interface provides results similar to those obtained for samples spotted using the traditional dried-drop technique. Moreover, the analysis of Trypsin digests of both BSA and Cytochrome C indicate that the adoption of the droplet-interface would efficiently conserve the resolution obtained by the upstream separation process. The reproducible generation of MALDI-ready droplets and their successful extraction from an oil stream, when combined with automated spotting, permits the deposition of single droplets and enables analysis of extremely complex peptide mixtures originating from many proteins.

## Supporting Information

Figure S1 (TIFF)

Figure S2 (TIFF)

Figure S3 (TIFF)

Table S1 (TIFF)

Table S2 (TIFF)

Table S3 (TIFF)

Table S4 (TIFF)

Video S1 (AVI)
