# Supplementary figures and images for: A Nano LC-MALDI Mass Spectrometry Droplet Interface for the Analysis of Complex Protein Samples
Source: PLoS One. 2013 May 9;8(5):e63087. doi: 10.1371/journal.pone.0063087 (PMC3650041; doi:10.1371/journal.pone.0063087)

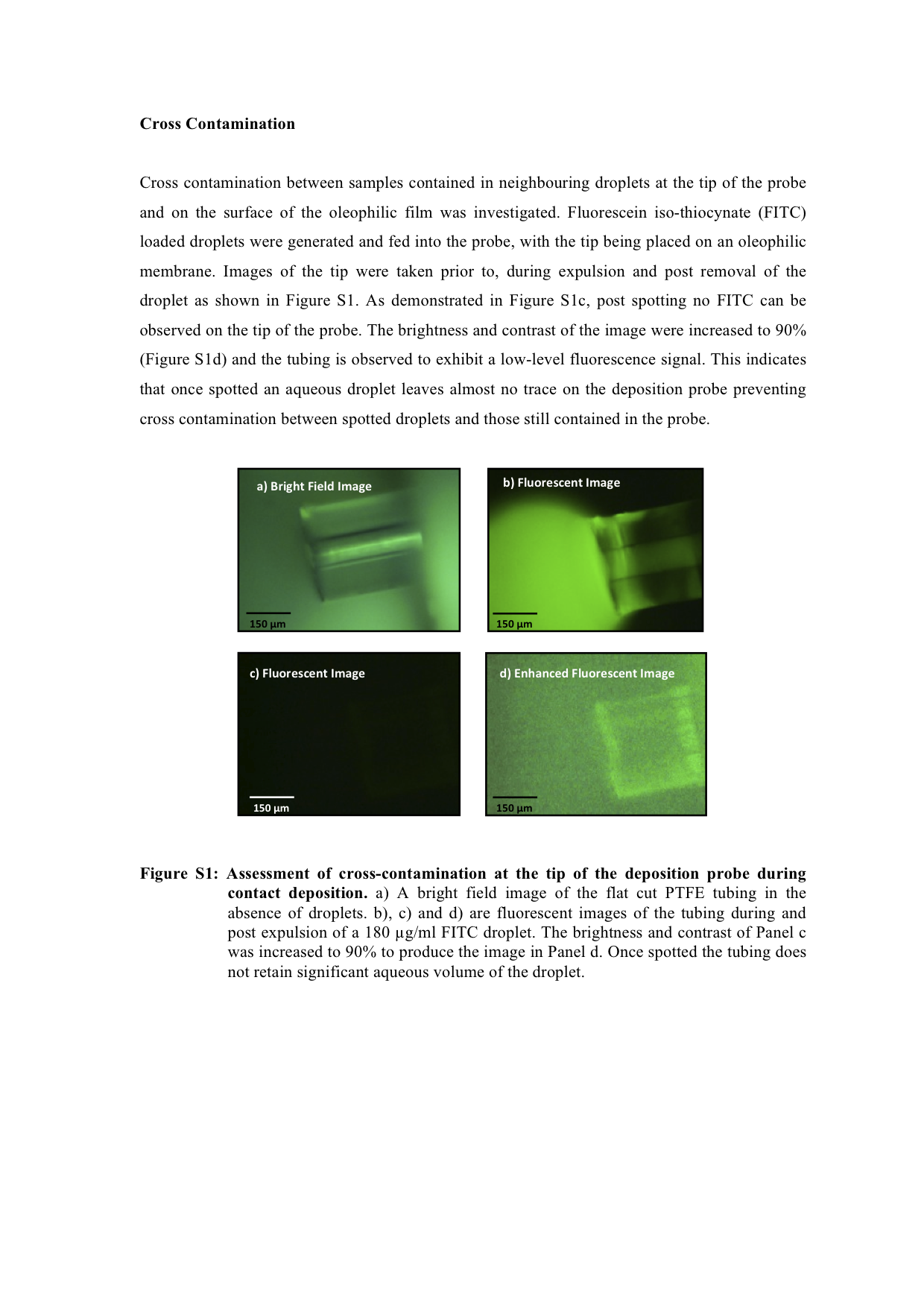

Supplement: Figure S1 — (TIFF) [file pone.0063087.s001.tiff]

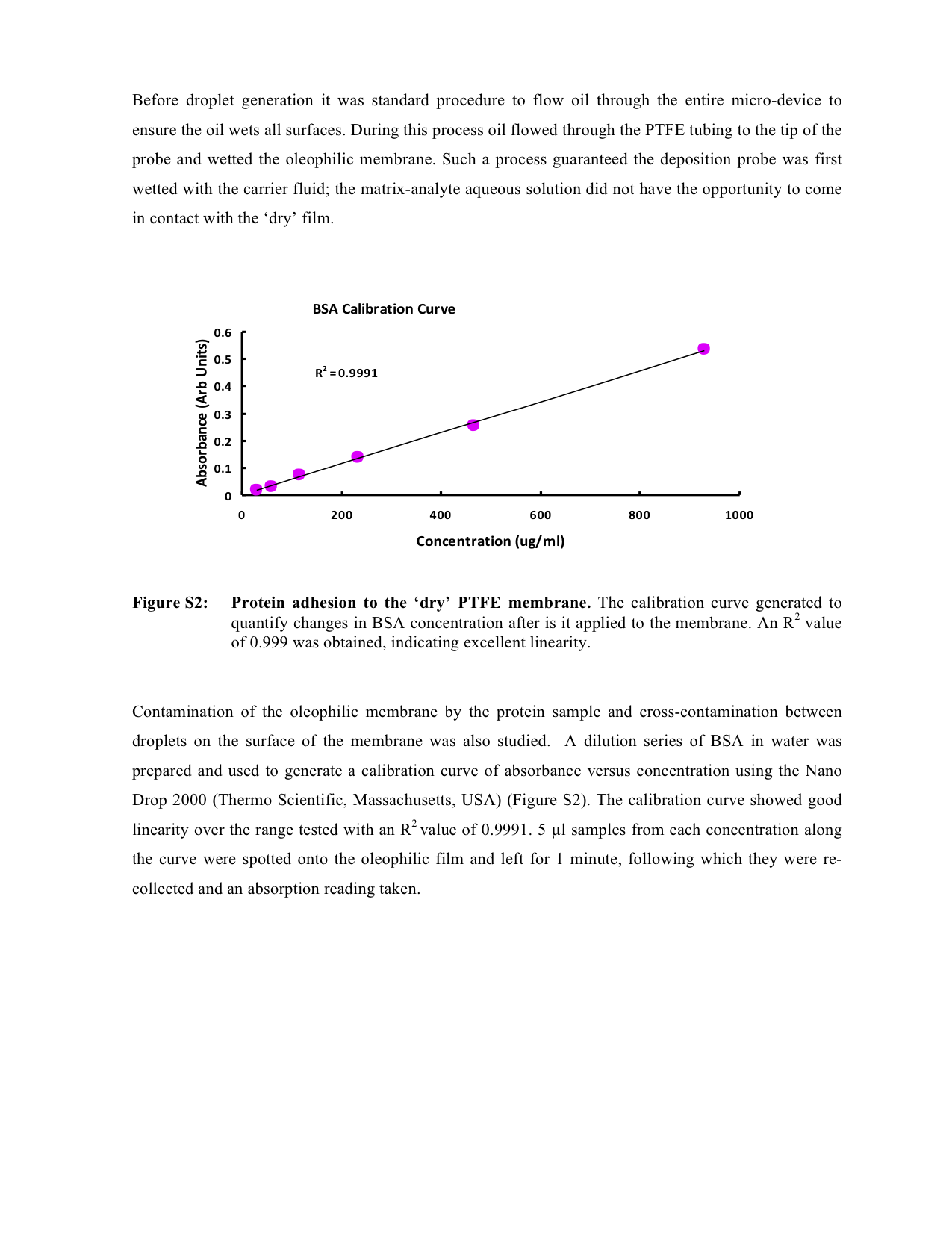

Supplement: Figure S2 — (TIFF) [file pone.0063087.s002.tiff]

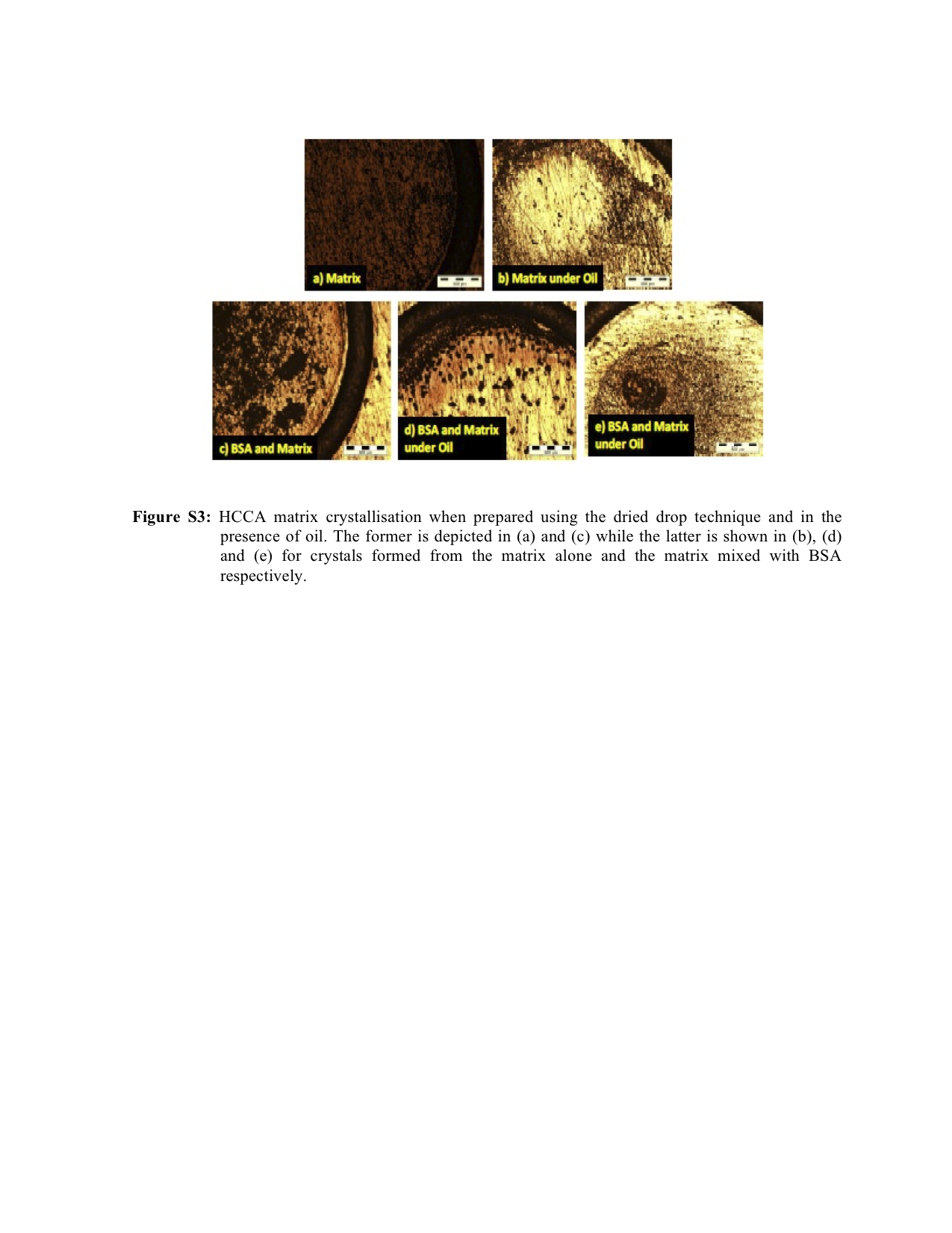

Supplement: Figure S3 — (TIFF) [file pone.0063087.s003.tiff]

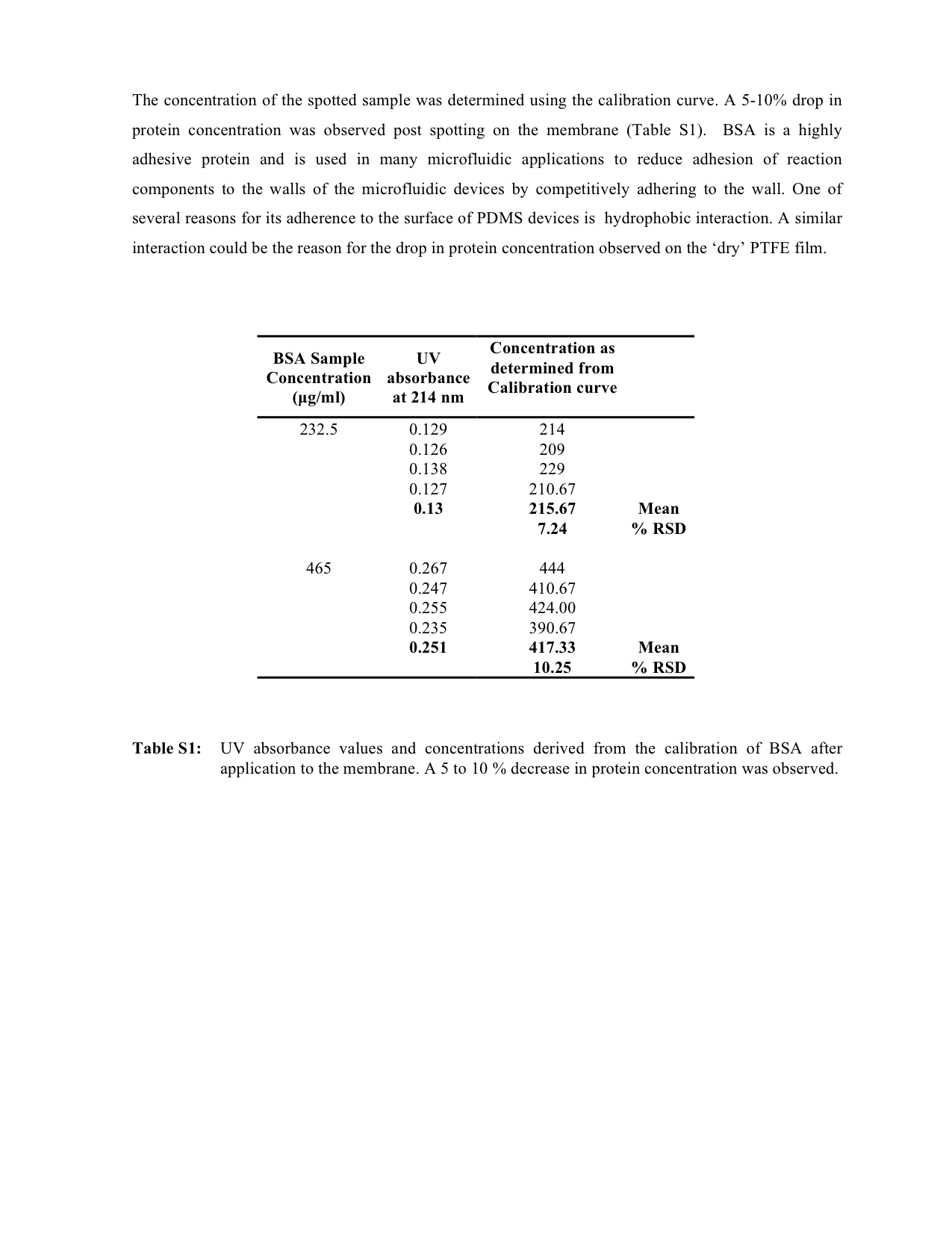

Supplement: Table S1 — (TIFF) [file pone.0063087.s004.tiff]

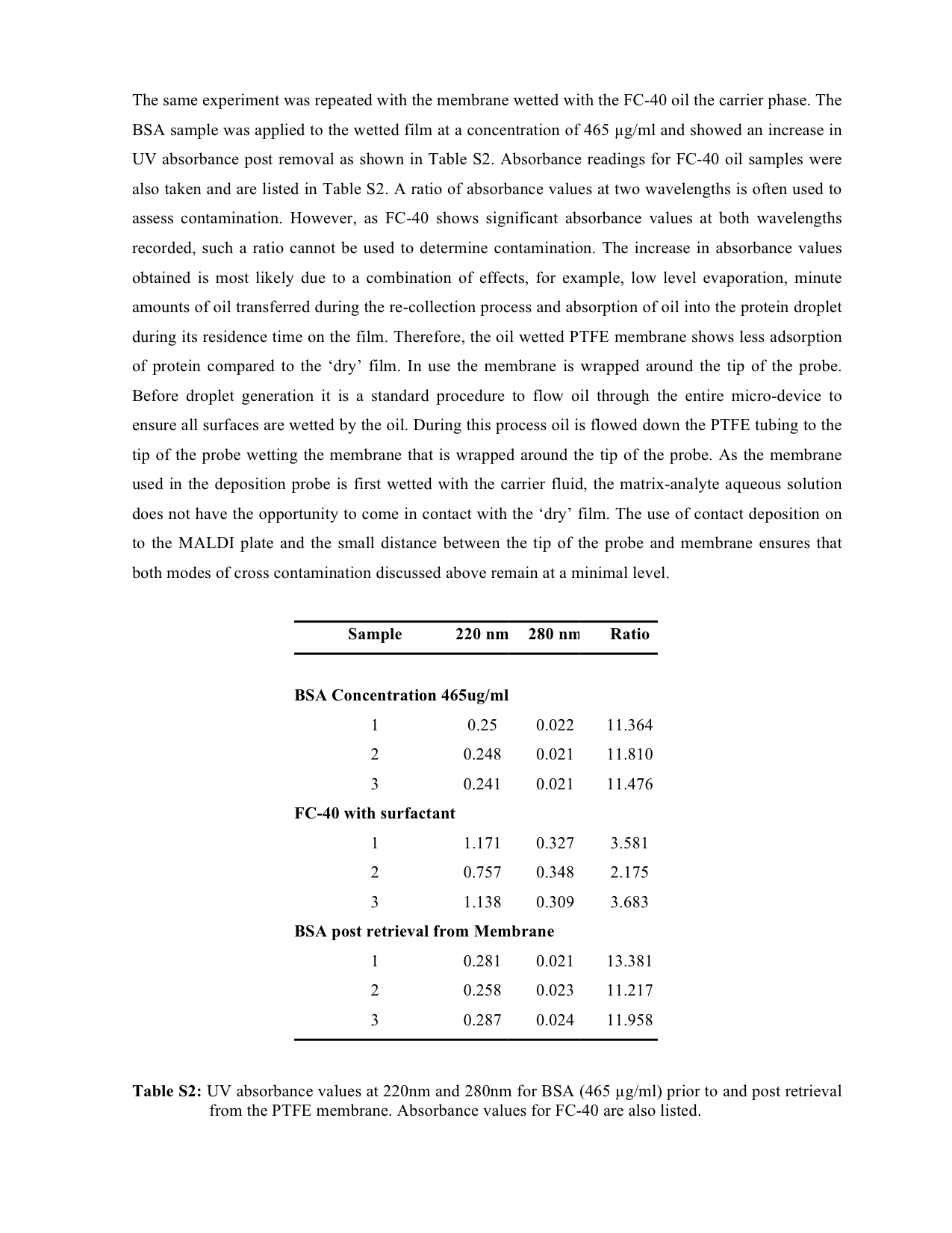

Supplement: Table S2 — (TIFF) [file pone.0063087.s005.tiff]

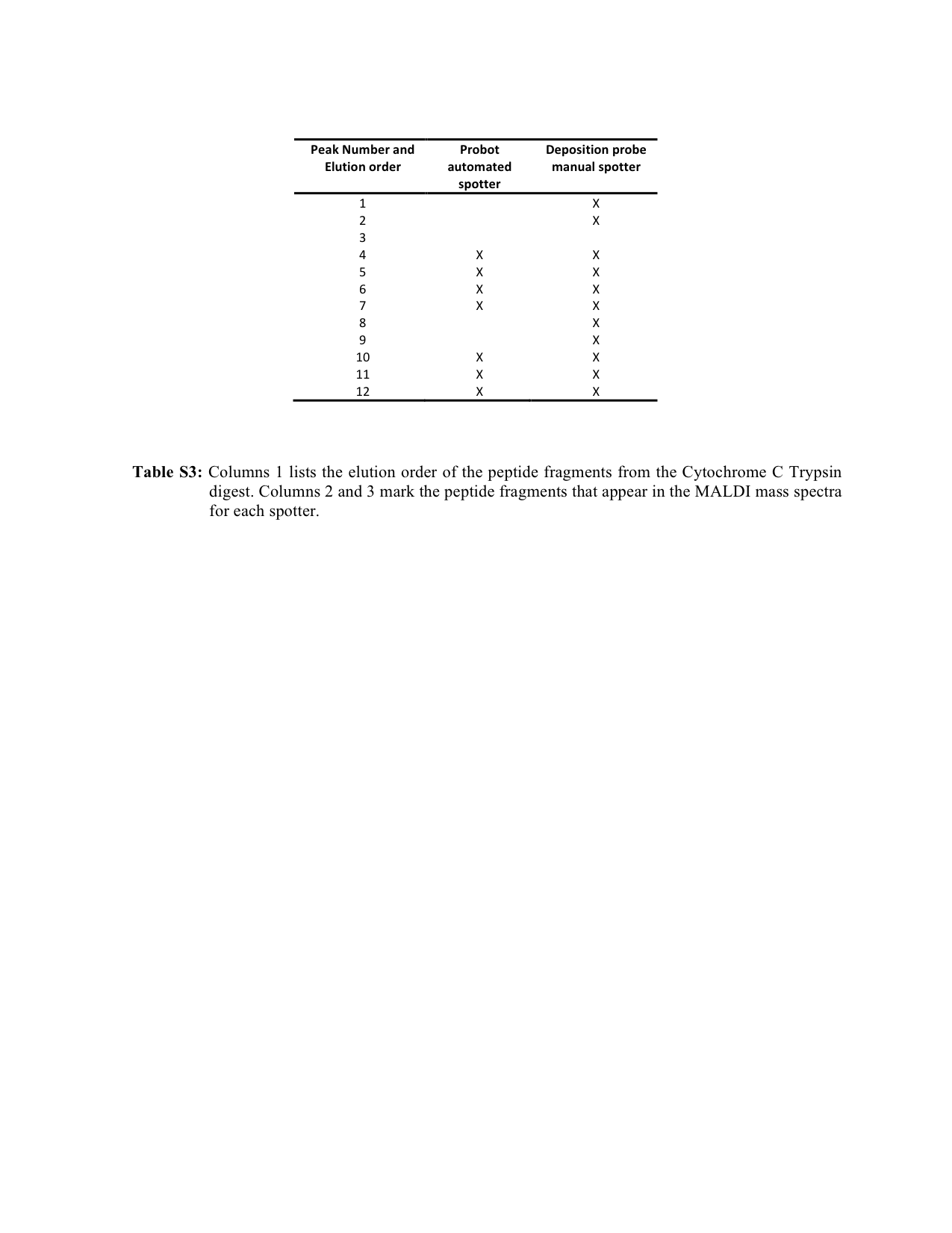

Supplement: Table S3 — (TIFF) [file pone.0063087.s006.tiff]

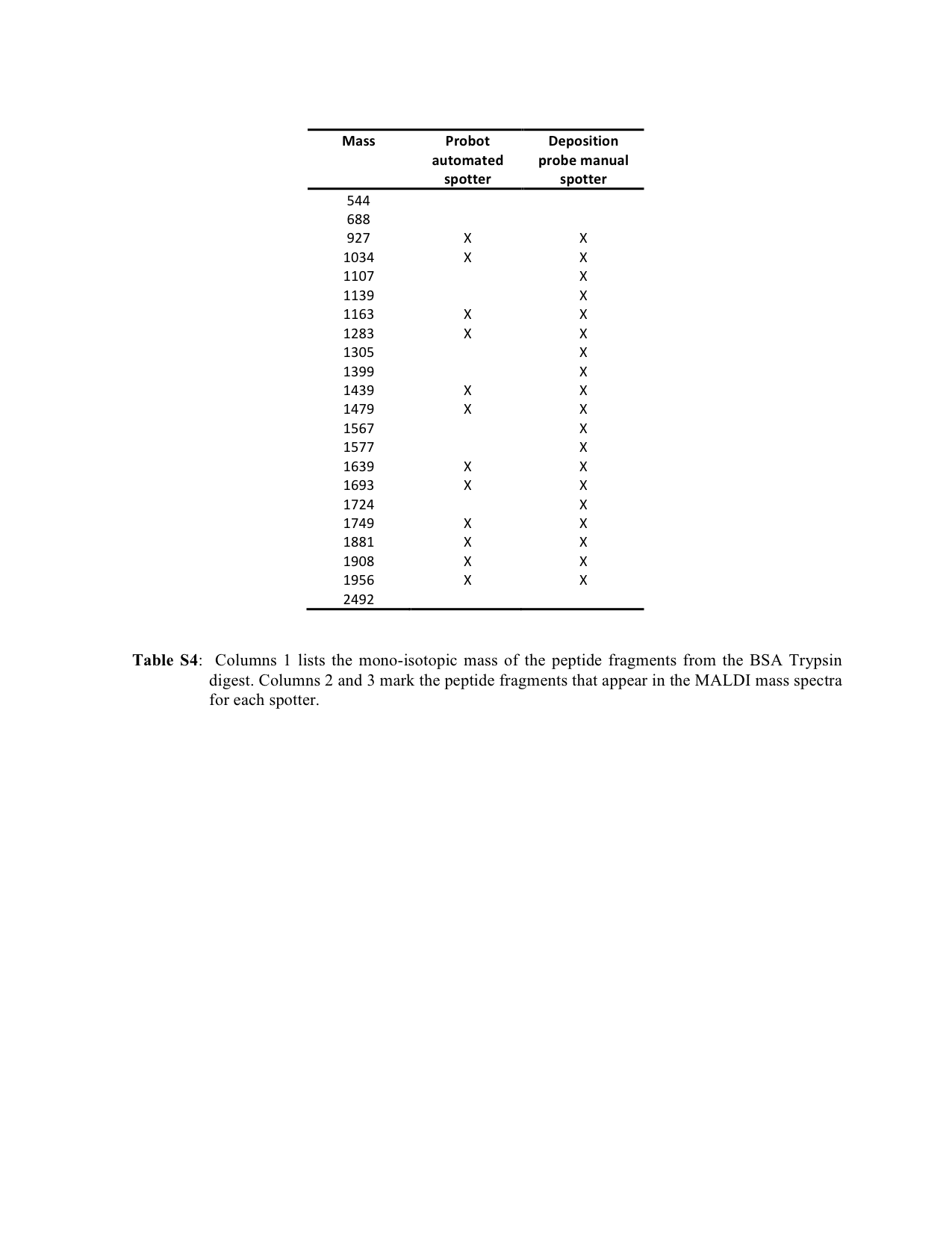

Supplement: Table S4 — (TIFF) [file pone.0063087.s007.tiff]
